# Changes in Touch Avoidance, Stress, and Anxiety During the COVID-19 Pandemic in Italy

**DOI:** 10.3389/fpsyg.2022.854110

**Published:** 2022-07-22

**Authors:** Marcello Passarelli, Laura Casetta, Luca Rizzi, Raffaella Perrella, Giuseppe Maniaci, Daniele La Barbera

**Affiliations:** ^1^Institute for Educational Technology, National Research Council of Italy, Genoa, Italy; ^2^Associazione Centro di Psicologia e Psicoterapia Funzionale, Istituto SIF di Padova, Padua, Italy; ^3^Department of Psychology, University of Campania “Luigi Vanvitelli,” Caserta, Italy; ^4^Department of Biomedicine, Neuroscience and Advanced Diagnostics, University of Palermo, Palermo, Italy

**Keywords:** touch avoidance, COVID-19, fear of contagion, pandemic effects, attitudes toward touch

## Abstract

In the present study we analyzed how attitudes toward touch have changed during the COVID-19 pandemic in an Italian sample, through two different studies: in the first we contacted participants of the Italian validation study of the Touch Avoidance Questionnaire, asking them to take part in a follow-up study (*N* = 31, 64.5% women, age 42.58 ± 15.15); in the second we recruited a new sample of 717 people (73.92% women, age 34.25 ± 13.11), comparing it to the full validation sample of the Touch Avoidance Questionnaire (*N* = 335, 64.48% women, age = 35.82 ± 14.32) to further investigate the relationship between the pandemic, stress responses, fear of contagion, anxiety, and attitudes toward touch. Overall, we found higher post-pandemic scores for touch avoidance toward strangers and family members and lower scores in touch avoidance toward friends of either gender, along with a slight increase in anxiety and stress. Touch avoidance was also positively related to anxiety and/or stress levels except for touch avoidance toward same-sex friends, for which the relationship with anxiety was negative. Surprisingly, we found that young people were the most anxious, despite older people being more at-risk of dying from COVID-19. Women were slightly more stressed out. COVID-19-related fears were significant predictors of touch avoidance toward partners, friends and strangers, but not of touch avoidance toward family. The results suggest that touch avoidance increased during the pandemic (except toward same-sex friends), together with anxiety and stress levels, but the change was relatively small.

## Introduction

The novel coronavirus SARS-CoV-2 emerged in December 2019, quickly spreading throughout the world. In Italy, one of the first nations hit by the pandemic, the government swiftly imposed a nation-wide lockdown and social distancing measures to contain the outbreak, lasting from March 9 to the beginning of May 2020. Partial lockdown measures, including social distancing, were imposed again in the autumn following the second outbreak.

During the pandemic, several studies focused on the psychological consequences of the pandemic and related prevention measures. An Italian study highlighted high rates of anxiety, distress, and symptoms related to post-traumatic stress disorder in a group of 2,291 respondents (Casagrande et al., 2020). During the second wave of contagion in Italy (November–December 2020), there was a strong increase in sleep disorders, which were related to depression, stress, and anxiety (Salfi et al., 2021a), although the prospect of a vaccination campaign did seem beneficial (Salfi et al., 2021b). Another study analyzed the effects of the lockdown period among Italian late adolescents and elderly people, finding that adolescents displayed higher levels of depression and perceived stress than older people (Amicucci et al., 2021).

Analyzing the international literature about the pandemic’s effects, one may argue that the psychological distress could be interpreted as the result of several factors: fear of contagion, loss of loved ones, financial insecurity, and feelings of isolation. The latter could be a consequence of social distancing measures, radically limited social interactions, especially physical closeness (Every-Palmer et al., 2020).

This study aims to investigate both how attitudes toward physical touch changed in response to the pandemic and how they relate to anxiety and stress, as the lack of physical touch may have had an impact on psychological wellbeing. Physical touch, a primary need for humans and other primates, is related to social bonding, feelings of safety (Dunbar, 2010) and, ultimately, wellbeing (Jakubiak and Feeney, 2019; Packheiser et al., 2021). Prior to the COVID-19 pandemic, social touch was one of the ways people regulated their stress, sought proximity to close others (especially romantic partners) and disclosed their problems and distress in an effort to regulate their emotions (Hazan and Shaver, 1987; Mikulincer et al., 2002; Zaki and Craig Williams, 2013). Moreover, touch seems to be especially important for fostering intimacy (App et al., 2011) and exhibiting love and sympathy (Hertenstein et al., 2009).

Social touch has been categorized into three types: (1) simple, described as a touch of short duration and applied on a restricted part of the body; (2) protracted, involving longer and mutual contact, such as hugging or holding hands; and (3) dynamic, comprising continuous and repetitive movements over the skin, such as caressing (Morrison et al., 2010; Vieira et al., 2016).

The physiological effects of social touch have been studied in recent decades. For example, daily hugging behaviors over a 14-day period have been shown to be significantly and inversely related to proinflammatory cytokines (van Raalte and Floyd, 2021). Similarly, in women, the reported frequency of hugs with partners is correlated with elevated oxytocin levels and lowered blood pressure (Light et al., 2005). Moreover, touch has been linked to lower blood pressure, lower heart rate, and higher oxytocin level (Field, 2010), which is itself related to lower levels of inflammation (Jankowski et al., 2010).

Despite the overall psychophysiological benefits of touch, contextual and personal factors influence each person’s attitude toward touching and being touched, and these could include the response to the pandemic.

Regarding contextual factors, receiving a gentle stroke from an undesirable toucher, for example, changes the pleasant experience into disgust (Ellingsen et al., 2016). Touching behavior is also an expression of dominance associated not only with social status, but also with gender, especially in countries where men are still in clearly dominant positions (Dibiase and Gunnoe, 2004). Additionally, attitude toward touch could be a general individual predisposition. Andersen and Leibowitz (1978) defined a person’s attitude toward touching and being touched in terms of touch avoidance (Ozolins and Sandberg, 2009; Russo et al., 2020). Touch avoidance is influenced by gender and by attachment processes built during infancy and adulthood and is a stable personality trait (Johansson, 2013; Ekeberg, 2016).

When considering individual attitudes toward touch, compared to men, women generally show more positive attitudes toward touch with friends and romantic partners and greater willingness to engage with such touch. However, women are more likely to perceive touch from opposite-sex strangers as unpleasant (Ozolins and Sandberg, 2009; Russo et al., 2020; Passarelli et al., 2021). Age is another factor influencing the pleasantness of touch: affective touch is perceived as more pleasant at a young age (Sehlstedt et al., 2016; Croy et al., 2019). Psychophysiological factors that influence touch avoidance include chronic pain, depression, and anhedonia, all conditions that reduce the hedonic experiences connected to affiliative touch (Pizzagalli et al., 2008; Elvemo et al., 2015; Rømer Thomsen, 2015). Lastly, anxiety seems to be related to touch avoidance: individuals with high trait anxiety appear to be more touch avoidant (Vieira et al., 2016), and social anxiety is accompanied by heightened aversion toward social situations involving touch (Wilhelm et al., 2001).

During the pandemic, social distancing was imposed in an effort “to decrease/interrupt transmission in a population by minimizing contact between potentially infected and healthy individuals” (ECDPC, 2020). These social distancing measures were mostly successful at first, but their emotional costs were not considered. During the COVID-19 pandemic, such policies prompted a change in the attitudes toward social touch, where culturally encouraged touch conveying care, affection and love such as hugging, hand-shaking or just sitting close together while comforting a dying relative was prohibited and labeled as “dangerous” for the transmission of the virus. One consequence of defining social touch as a way to convey the contagion was that it reframed a behavior associated with care and love to a possible threat to oneself and to loved ones (Green and Moran, 2021).

The forced behavioral changes toward touch during the pandemic may have deprived people of a main strategy for managing their distress; it also may have a long-term influence on individual attitudes toward touch by reframing social touch as a dangerous behavior. However, our current understanding of the pandemic’s dynamics does not address the degree to which fear of contagion has changed the individual meaning that people attribute to social touch and whether this possible change influences people’s psychological wellbeing.

Given these premises, we aimed to investigate three different research questions (RQs): (RQ1) whether the COVID-19 pandemic increased touch avoidance (i.e., personal aversion toward touch) in a sample of Italian subjects; (RQ2) how touch avoidance is related to anxiety and distress; and (RQ3) whether touch avoidance is associated with COVID-related fears and risk factors for COVID-19 (i.e., being male, being over 60 years old, being unvaccinated, knowing someone who died from COVID-19).

Those aims were examined in two different studies. The first one took advantage of the sample recruited by Casetta et al. (2020) for the validation of a touch avoidance measure conducted just prior to the pandemic. Re-recruiting some participants from the study allowed us to directly compare attitudes toward touch in a pre-post study design. This study focuses on RQ1 and RQ2, but the small sample size does not allow us to investigate RQ3.

Research questions 1–3 were investigated in Study 2, which recruited a larger sample toward the end of the second Italian outbreak by proposing an online survey. These data were analyzed in comparison with the full Touch Avoidance Questionnaire validation sample, which also measured anxiety and stress prior to the pandemic.

## Study 1

### Methods

#### Participants and Procedure

For this study, we contacted participants from the Italian validation study of the Touch Avoidance Questionnaire (TAQ; Casetta et al., 2020), asking them if they wanted to take part in this follow-up study. Only participants who expressly reported interest in participating in further studies were contacted, and 31 participants (20 women, 64.5%; age 42.58 ± 15.15) responded positively. The data for the TAQ validation were collected in the first half of 2019, prior to the COVID-19 pandemic (protocol approved by the Ethics Committee of the University of Campania “Luigi Vanvitelli,” Department of Psychology, 04/2019). While the pre-pandemic data collection was performed using paper-and-pencil questionnaires, the second round was conducted online using Google Forms due to social distancing measures. After providing informed consent and some socio-biographical information such as age, gender, and educational level, participants were asked a series of questions regarding the context in which they experienced the COVID-19 pandemic (e.g., whether they knew someone who died from COVID-19, whether they lived alone during the lockdown, whether they worked remotely, etc.). Afterward, they completed a series of questionnaires described in the following section. The Ethics Committee of Policlinico “Paolo Giaccone” in Palermo, Italy, approved this second round of data collection (06/2020). Recruiting and testing conformed with the local Ethics Committee requirements and the Declaration of Helsinki.

#### Measures

##### Touch Avoidance Questionnaire

The TAQ (Ozolins and Sandberg, 2009; Casetta et al., 2020) is a 37-items questionnaire that investigates attitudes toward touch. Items are responded to on a 5-point scale ranging from 1 (“fully disagree”) to 5 (“fully agree”). The questionnaire comprises 5 subscales, corresponding to 5 different categories of individuals with whom touch could be experienced: romantic partners (10 items), same-sex friends (6 items), opposite-sex friends (6 items), family (6 items), and strangers (3 items). For all subscales, higher scores indicate higher touch avoidance (i.e., aversion to touch). The Italian version of the scale has an ordinal alpha ranging 0.59–0.92 for the five subscales and 1-month retest reliability ranging 0.67–0.90. The “stranger” subscale has a lower alpha (0.59), partly due to the low number of items in it, but high test-retest correlation (0.82 after 1 month in the Italian validation study).

##### Mesure de Stress Psychologique

The Mesure de Stress Psychologique (MSP) (Lemyre and Tessier, 1988; Di Nuovo et al., 2000) is a 49-item self-report questionnaire that measures responses to stress. The participant is asked to self-evaluate how much they felt, in the last 4–5 days, some typical stress responses (e.g., “I feel fatigued,” “I cry easily,” or “I take more than half an hour to fall asleep”). Items consider thoughts, somatic symptoms, emotions, and behaviors. The response scale for each item ranges from 1 (“not at all”) to 4 (“to a great extent”). Higher scores indicate higher levels of stress responses. The Cronbach’s alpha for the scale is 0.94.

##### State-Trait Anxiety Inventory

The State-Trait Anxiety Inventory (STAI-Y) (Pedrabissi and Santinello, 1989) is a 40-item questionnaire investigating state and trait anxiety. The response scale for each item ranges from 1 (“not at all”) to 4 (“extremely”). Higher scores indicate higher levels of anxiety. In this study, we only included the state anxiety subscale, which has Chronbach’s alpha of 0.93.

#### Statistical Analysis

Analyses were conducted using R 4.0.2. Missing data (0.2%) were treated using pairwise deletion. When performing multiple tests, *p*-values were corrected for multiple comparisons using Benjamini-Hochberg’s correction (Benjamini and Hochberg, 1995). All tests were two-tailed.

RQ1 was examined by Wilcoxon signed-rank tests comparing (sub)scale scores before and during the pandemic. RQ2 was investigated using a single maximum likelihood path analysis in which gender, age and the post-pre difference on TAQ scores simultaneously predict STAI and MSP scores during the pandemic.

R codes are available at the following repository: https://github.com/M-Pass/TouchAvoidance_Covid/blob/main/Study1.R.

### Results

#### RQ1

Descriptive statistics and a correlogram of (sub)scale scores are available in the Supplementary Material. Results for the Wilcoxon signed-rank tests are reported in Table 1.

**TABLE 1:** Pre-post comparisons for the scores on each subscale.

(Sub)scale	Difference (post—pre)	*V*	*P*	*Cohen’s d*
TAQ partner	0.01	195	0.542	0.01 (negligible)
TAQ family	−0.07	138	0.392	0.07 (negligible)
TAQ same sex	0.23	185	0.392	0.34 (small)
TAQ opposite sex	0.05	209	0.900	0.07 (negligible)
TAQ stranger	0.78	315	<0.001***	1.02 (large)
MSP	−0.12	178	0.392	0.27 (negligible)
STAI-Y1	−0.02	192	0.900	0.05 (negligible)

***significant for p < 0.001. All p-values are adjusted for multiple comparisons using Benjamini-Hochberg’s correction.

The only significant difference was in touch avoidance toward strangers, which greatly increased during the pandemic. Touch avoidance toward other categories of people was unchanged, although the effect size for touch avoidance toward same-sex friends was not negligible, suggesting that a study involving a larger sample may find an effect.

#### RQ2

Results are reported in Table 2. *R*^2^ for the MSP is 0.33; *R*^2^ for the STAI-Y1 is 0.27. The residual correlation between MSP and STAI-Y1 scores is 0.74.

**TABLE 2:** Path analysis predicting MSP and STAI-Y1 scores.

Scale	Predictor	β	*Z*	*P*
MSP (Post)	Age	−0.49	−2.78	0.005**
	Gender (male)	0.00	0.01	0.995
	TAQ partner	−0.08	−0.33	0.741
	TAQ family	−0.03	−0.11	0.911
	TAQ same sex	−0.35	−1.72	0.086
	TAQ opposite sex	0.26	1.21	0.228
	TAQ stranger	−0.33	−1.90	0.057
STAI-Y1 (Post)	Age	−0.47	−2.56	0.010*
	Gender (male)	−0.08	−0.45	0.656
	TAQ partner	0.08	0.31	0.758
	TAQ family	0.02	0.07	0.947
	TAQ same sex	−0.01	−0.03	0.973
	TAQ opposite sex	−0.15	−0.66	0.512
	TAQ stranger	−0.26	−1.46	0.144

*Significant for p < 0.05, **significant for p < 0.01.

The only significant predictor was age, as older people had, on average, less stress and anxiety compared to younger individuals. No other predictor was significant, suggesting that both stress and anxiety are unrelated to change in attitudes toward social touch. However, as the small sample size would make any interpretation of these results tentative, we ran a larger-scale study to further investigate the relationship between the pandemic, stress responses, anxiety, and touch avoidance.

## Study 2

### Methods

#### Participants and Procedure

In this study, a new sample of participants took part in a single data collection conducted from April to May 2021 (a period in which Italy was still experiencing a peak of infections and travel restrictions and curfew were still in place on a nation-wide level). Participants were recruited online through snowball sampling (Parker et al., 2019). After providing informed consent, participants were asked to complete the same materials described in Study 1 (i.e., socio-biographical information, COVID-related questions, TAQ, MSP, STAI-Y1), as well as the Multidimensional Assessment of COVID-19—Related Fears (MAC-RF; see below). A total of 717 individuals took part in the study (530 women, 73.92%; 182 men, 25.38%; 5 undisclosed, 0.42%; age 34.25 ± 13.11; range, 18–78). The sample was unevenly distributed geographically (35% from North Italy, 7% from central Italy, 58% from South Italy/islands). Data collected from this sample were compared with data collected for the Italian validation of the TAQ (Casetta et al., 2020; *N* = 335, 216 women, 64.48%; 113 men, 33.63%; 6 undisclosed, 1.79%; age = 35.82 ± 14.32; range, 16--74; 35% from North Italy, 35% from central Italy, and 31% from South Italy/islands).^1^ The two samples differed in gender composition [X 2(1) = 8.12, *p* = 0.004], with the validation (pre-COVID) sample having significantly fewer women than the one collected for this study. The age difference was not significant [*t*(595.55) = 1.68, *p* = 0.093]. The Ethics Committee of Policlinico “Paolo Giaccone” in Palermo, Italy, approved this study (02/2021). Recruiting and testing conformed with the local Ethics Committee requirements and the Declaration of Helsinki.

#### Measures

##### Multidimensional Assessment of COVID-19—Related Fears

In addition to the measures employed in Study 1, this survey included the Multidimensional Assessment of COVID-19—Related Fears (MAC-RF) (Schimmenti et al., 2020). This test is an eight-item, self-report scale that has been developed to assess clinically relevant domains of fear during the COVID-19 pandemic. The MAC-RF is based on a comprehensive theoretical model conceptualizing fears during the pandemics as resulting from an interaction of bodily, interpersonal, cognitive, and behavioral experiences. The authors have suggested four dialectical elements of COVID-19 fear: fear of and for one’s body, as one is both a potential vector and victim; fear of and for others, also related to the tension of prescribed social distancing from important interpersonal relationship; fear of ignorance of the virus as well as knowledge, as information is required for protection but can also be overwhelming and anxiety-inducing; and fear of both personal action and inaction, related to the behavioral consequences of fear. Respondents rate all 8 items on a 5-point Likert scale (from 0 = very unlike me to 4 = very like me). By adding all items’ rates, a total score can be obtained. The higher it is, the stronger COVID-19 related fears are. Cronbach’s α for the whole scale is 0.77.

#### Statistical Analysis

Analyses were conducted using R 4.0.2. Missing data (1.4% for the pre-pandemic sample, 0.5% for the pandemic sample) were treated using pairwise deletion. When performing multiple tests, *p*-values were corrected for multiple comparisons using Benjamini-Hochberg’s correction (Benjamini and Hochberg, 1995). All tests were two-tailed.

RQ1 was examined with independent samples *t*-tests comparing (sub)scale scores between the samples collected before and during the pandemic. RQ2 and RQ3 were investigated using a maximum likelihood path analysis in which COVID-related fears and COVID risk factors (gender, being over 60, being vaccinated and knowing people who died from COVID-19) predict TAQ, MSP and STAI scores and in which TAQ scores are used in turn as predictors of MSP and STAI scores (although actual causality could be reversed or even bidirectional). Results for RQ2 and RQ3 are presented separately, but they refer to the same model.

R codes are available at the following repository: https://github.com/M-Pass/TouchAvoidance_Covid/blob/main/Study2.R.

### Results

#### RQ1

Descriptive statistics and correlograms for all (sub)scale scores before and during the pandemic are reported in the Supplementary Material. Results for the independent samples *t*-tests are reported in Table 3.

**TABLE 3:** Pre-post comparisons for the scores on each subscale.

(Sub)scale	Pre-COVID score	Post-COVID score	*t*	*df*	*P*	*Cohen’s d*
TAQ partner	1.94	1.94	0.05	747.45	0.959	0.00 (negligible)
TAQ family	2.65	2.86	−3.24	679.52	<0.001***	0.21 (small)
TAQ same sex	2.07	1.92	2.96	725.95	<0.003**	0.19 (negligible)
TAQ opposite sex	2.16	2.02	2.63	744.98	0.008**	0.17 (negligible)
TAQ stranger	2.62	2.83	−3.66	715.88	<0.001***	0.23 (small)
MSP	1.89	2.13	−7.04	767.82	<0.001***	0.44 (small)
STAI-Y1	2.60	2.77	−4.67	765.46	<0.001***	0.29 (small)

**significant for p < 0.01, ***significant for p < 0.001.

The findings show higher post-pandemic scores for stress, anxiety and touch avoidance toward family members and strangers. However, the results indicate lower scores in touch avoidance toward friends of either gender. No change was found for touch avoidance toward romantic partners. All effect sizes were small or negligible, suggesting that while the pandemic affected attitudes toward touch, stress, and anxiety, the effect was very limited.

#### RQ2

Results are reported in Table 4. *R*^2^ was 0.35 for the MSP and 0.34 for the STAI-Y1. Residual correlation between the two measures is 0.167.

**TABLE 4:** Path analysis predicting MSP and STAI-Y1 scores.

Scale	Predictor	β	*z*	*P*
MSP	Age (Over 60)	−0.10	−2.95	0.003**
	Gender (Male)	−0.08	−2.16	0.031*
	Vaccinated (Yes)	−0.01	−0.43	0.667
	Knows people who died from COVID-19 (Yes)	0.05	1.40	0.162
	TAQ partner	0.09	2.62	0.009**
	TAQ family	0.16	4.57	<0.001***
	TAQ same sex	−0.08	−1.77	0.077
	TAQ opposite sex	0.03	0.64	0.522
	TAQ stranger	0.11	2.86	0.004**
	MAC-RF1 (Fears related to the body)	0.08	1.76	0.079
	MAC-RF2 (Fears related to meaningful relationships)	0.00	0.13	0.897
	MAC-RF3 (Difficulties in cognitive monitoring of concerns)	0.12	2.96	0.003**
	MAC-RF4 (Behavioral difficulties related to fear)	0.37	8.39	<0.001***
STAI-Y1	Age (Over 60)	−0.09	−2.74	0.006**
	Gender (Male)	0.03	0.91	0.365
	Vaccinated (Yes)	−0.03	−0.80	0.426
	Knows people who died from COVID-19 (Yes)	−0.04	−1.30	0.194
	TAQ partner	0.13	3.61	<0.001***
	TAQ family	0.15	4.31	<0.001***
	TAQ same sex	−0.15	−3.40	0.001**
	TAQ opposite sex	0.11	2.50	0.012*
	TAQ stranger	0.10	2.65	0.008**
	MAC-RF1 (Fears related to the body)	0.00	−0.05	0.957
	MAC-RF2 (Fears related to meaningful relationships)	0.13	2.72	0.007**
	MAC-RF3 (Difficulties in cognitive monitoring of concerns)	0.07	1.69	0.091
	MAC-RF4 (Behavioral difficulties related to fear)	0.36	8.25	<0.001***
TAQ partner	Gender (Male)	−0.00	−0.09	0.928
	Age (Over 60)	0.08	1.94	0.052
	Vaccinated (Yes)	−0.08	−1.97	0.049*
	Knows people who died from COVID-19 (Yes)	0.01	1.16	0.871
	MAC-RF1 (Fears related to the body)	0.01	0.22	0.829
	MAC-RF2 (Fears related to meaningful relationships)	−0.12	−2.17	0.030*
	MAC-RF3 (Difficulties in cognitive monitoring of concerns)	0.05	0.94	0.348
	MAC-RF4 (Behavioral difficulties related to fear)	0.09	1.76	0.078
TAQ family	Gender (Male)	0.09	2.29	0.022*
	Age (Over 60)	0.04	0.97	0.334
	Vaccinated (Yes)	−0.10	−2.35	0.019*
	Knows people who died from COVID-19 (Yes)	0.04	1.03	0.302
	MAC-RF1 (Fears related to the body)	−0.01	−0.22	0.824
	MAC-RF2 (Fears related to meaningful relationships)	−0.06	−1.10	0.272
	MAC-RF3 (Difficulties in cognitive monitoring of concerns)	0.02	0.33	0.740
	MAC-RF4 (Behavioral difficulties related to fear)	0.05	0.99	0.320
TAQ same Sex	Gender (Male)	0.25	6.36	<0.001***
	Age (Over 60)	0.13	3.27	0.001**
	Vaccinated (Yes)	0.00	0.01	0.992
	Knows people who died from COVID-19 (Yes)	0.00	0.06	0.952
	MAC-RF1 (Fears related to the body)	0.07	1.25	0.213
	MAC-RF2 (Fears related to meaningful relationships)	−0.05	−0.94	0.347
	MAC-RF3 (Difficulties in cognitive monitoring of concerns)	0.09	1.87	0.062
	MAC-RF4 (Behavioral difficulties related to fear)	0.11	2.22	0.026*
TAQ opposite sex	Gender (Male)	−0.06	−1.53	0.127
	Age (Over 60)	0.12	2.97	0.003**
	Vaccinated (Yes)	−0.04	−0.90	0.371
	Knows people who died from COVID-19 (Yes)	0.04	1.14	0.253
	MAC-RF1 (Fears related to the body)	0.07	1.27	0.205
	MAC-RF2 (Fears related to meaningful relationships)	−0.09	−1.65	0.099
	MAC-RF3 (Difficulties in cognitive monitoring of concerns)	0.14	2.88	0.004**
	MAC-RF4 (Behavioral difficulties related to fear)	0.12	2.24	0.025*
TAQ stranger	Gender (Male)	0.03	0.69	0.493
	Age (Over 60)	0.02	0.39	0.697
	Vaccinated (Yes)	0.01	0.26	0.794
	Knows people who died from COVID-19 (Yes)	0.01	0.23	0.821
	MAC-RF1 (Fears related to the body)	0.13	2.31	0.021*
	MAC-RF2 (Fears related to meaningful relationships)	0.12	2.09	0.037*
	MAC-RF3 (Difficulties in cognitive monitoring of concerns)	0.08	1.57	0.116
	MAC-RF4 (Behavioral difficulties related to fear)	0.03	0.57	0.572

*Significant for p < 0.05, **significant for p < 0.01, ***significant for p < 0.001.

Males presented lower stress (MSP) scores. Both STAI-Y1 and MSP scores were lower for participants over 60 years old and were positively related with touch avoidance toward romantic partners, family, and strangers. Additionally, STAI scores were related to both same-sex and opposite-sex touch avoidance, although with contrasting signs, as the relationship between same-sex touch avoidance and anxiety is negative. Regarding COVID-related fears, behavioral difficulties were found to correlate with both anxiety and stress, while difficulties in cognitive monitoring of concerns correlated with distress, but not anxiety. Lastly, fears related to meaningful relationships correlated with anxiety, but not distress.

#### RQ3

Results are reported in Table 4. *R*^2^ was 0.024 for TAQ partner, 0.027 for TAQ family, 0.109 for TAQ same sex, 0.067 for TAQ opposite sex, and 0.081 for TAQ stranger.

According to the results, individuals over 60 had higher touch avoidance scores toward friends of either gender (and a difference in touch avoidance toward partners bordering on significance). Male participants had higher touch avoidance toward family and same-sex friends. Vaccinated individuals had lower touch avoidance toward partners and family members, although touch avoidance toward friends and strangers was the same for vaccinated and unvaccinated individuals. Lastly, COVID-related fears seem to have different associations with the measures of touch avoidance: fears related to the body and meaningful relationships affected touch avoidance toward strangers, difficulties in monitoring cognitive concerns were correlated with touch avoidance toward opposite-sex friends and behavioral difficulties were correlated with touch avoidance toward friends of any gender. Lastly, fears related to meaningful relationships were negatively correlated with touch avoidance toward partners.

## Discussion

While several studies have been conducted on general wellbeing during the pandemic, to the best of our knowledge, none have focused on how social distancing changed people’s attitudes toward touch and its psychological consequences. The aim of this study was to analyze how attitudes toward touch have changed in the last year due to a strong natural stressor like the COVID-19 pandemic, which reconceptualized social touch as a threat. We investigated this question through two different studies. In the first, we contacted participants from the Italian validation study of the Touch Avoidance Questionnaire (TAQ; Casetta et al., 2020), asking them to take part in a follow-up study. In the second, we recruited a new sample of 717 people and compared results to the full validation sample of the TAQ (*N* = 335) to further investigate the relationship between the pandemic, stress responses, anxiety, and attitudes toward touch.

The first study revealed a change in attitudes toward touch involving strangers, while the larger sample of the second study highlighted a significant change in touch avoidance for family members (higher post-pandemic) and friends of either gender (lower post-pandemic), with slightly higher post-pandemic scores in the subscale measuring touch avoidance for strangers. These results suggest that the reconceptualization of touch as a source of contagion plays a role in the change of attitudes toward touch. Specifically, the higher touch avoidance toward family members could be related to fear of contaminating loved ones. Conversely, touch avoidance toward strangers could be due to the fear of being contaminated by others. The lower scores for touch avoidance toward friends during the pandemic are somewhat surprising, and may suggest a need to compensate for touch deprivation by getting physically closer to peer friends, an explanation consistent with the use of touch as an important way to deal with stress and to satisfy the need for connection (Baumeister and Leary, 1995). In accordance with a previous study (Passarelli et al., 2021), we found that, compared to men, women behave differently in relation to touch, being less touch avoidant toward same-sex friends (Ozolins and Sandberg, 2009; Hielscher and Mahar, 2017; Casetta et al., 2020). Their inclination to help others and to use social support to cope with negative emotions (Matud, 2004; Eisenbarth, 2019) is a reasonable explanation of the further decrease in women’s touch avoidance for same-sex friends during the pandemic. All changes in attitudes toward touch during the pandemic seem to be significant but small, suggesting that the behavioral changes forced by social distancing measures only had a limited impact on internal attitudes.

We then analyzed, in both data sets, how touch avoidance relates to stress and anxiety, finding that touch avoidance toward romantic partners and family members was associated with higher anxiety and stress levels, while touch avoidance toward strangers was only associated with stress, and touch avoidance toward opposite-sex friends was associated only with anxiety. All relationships were in the direction that suggested that touch avoidant individuals experience more stress/anxiety, with touch avoidance toward same-sex friends as the sole exception (for which the relationship was very weak, but negative). One possible interpretation of this result is that people who use social touch as a strategy to manage stress could be negatively affected by social distancing. However, it could also mean that anxious individuals—more affected by fear of contagion—tend to avoid social touch during a pandemic. Therefore, causality could be bidirectional. Differently from other research who highlighted a higher level of touch avoidance in elders (Ozolins and Sandberg, 2009) in this study the older people responded to the pandemic and social distancing with less anxiety and touch avoidance. This unexpected result can contribute to the field of social touch because highlights the importance of touch in managing stress independently from the fear of contagion. Even if elders are the most vulnerable category at risk of developing severe COVID related symptoms (Leung, 2020), our study has shown that the longing for social touch can help to go beyond the fear of contagion.

The reason for this phenomenon could be related to a greater wisdom and acceptance regarding death (Wysokiński et al., 2019), but further investigation is necessary to answer this question. Additionally, the fact that anxiety is higher for younger individuals may suggest that it relates more to spreading the contagion to loved ones, rather than fear for one’s own health.

To further understand the relationship between fear of contamination and touch avoidance, we analyzed whether COVID-related fears, as well as some risk factors for COVID-19 (namely age, gender, being vaccinated and knowing someone who died of COVID-19), influenced attitudes toward touch. The results suggest that, overall, older people and males are more touch avoidant, which is consistent with the hypothesis that touch avoidance correlates with risk of dying from COVID-19. However, we found no difference according to risk factors for touch avoidance toward strangers, arguably the most important source of contagion, which would disconfirm this hypothesis. The inconsistency of these results should be further investigated. Regarding COVID-related fears, they mostly influenced touch avoidance toward strangers and friends, while no significant effect was found for touch avoidance toward family. Specifically, we found a relation between the fear of being either a vector or victim of contagion and touch avoidance toward strangers. This could be interpreted as a sense of one’s physical vulnerability, causing the body to be perceived as a potential source of danger and strangers to be viewed as the most relevant threat of contagion. On the other hand, while fears related to meaningful relationships were negatively related to touch avoidance toward partners, fear of knowing important information was positively related to touch avoidance toward opposite-sex friends. Moreover, behavioral consequences of fear were positively correlated with touch avoidance toward friends of any gender. These results generally support the notion that COVID-related fears lead to touch avoidance. The one relationship for which individuals higher in COVID-related fears expressed lower touch avoidance is the relationship between fears related to meaningful relationship and touch avoidance toward partners. This result may relate to the use of intimate—and possibly sensual—touch as a way to cope with negative emotions.

Lastly, being vaccinated is associated with lower touch avoidance toward family and partners, suggesting that change in attitudes toward touch—which was already relatively small—could be partially reversible when the risk of contagion is lowered.

Summing up, from our study we may state that the pandemic and consequent social distancing has changed the Italian attitude toward touch only slightly: while the difference in touch avoidance before and during the pandemic was statistically significant for most subscales, it was smaller than we expected, suggesting that the pandemic, which changed behavior substantially, did not have much impact on underlying attitudes. This could suggest both that the Italian population was resilient to the changes in their lives due to the pandemic, and that the need for touch, being of prime importance for humans, cannot be entirely disregarded due to fear of contamination. The fact that being vaccinated reduced touch avoidance suggests that the changes in attitudes we observed may be not only small, but also temporary.

This study has several limitations. The sample size of the first study is quite small, and interpretation of its results should be tentative. Nevertheless, its longitudinal nature, with data collected prior to the appearance of COVID-19, can offer insights through data collected in circumstances that unfortunately cannot be replicated. Regarding the limitations of Study 1 and Study 2, having a single measurement during the pandemic does not allow us to investigate whether the apparent increase in psychological distress, anxiety, and touch avoidance was sustained over time. The third limitation is that to answer the survey, it was necessary to have access to a computer or internet-connected mobile phone and to be able to use them, so we probably over-represented people with higher socio-economic status and education, as well as the younger population. Hence, selection bias may have influenced our results: many respondents are young and female. Despite the methodological limitations, this study suggests that attitudes toward touch have changed—if only slightly—due to a strong natural stressor such as the COVID-19 pandemic. However, results suggest that the Italian population was psychologically resilient to the effects of lockdown and social distancing (Prati and Mancini, 2021).

We hope these data may be taken into consideration by political organizations and public health providers to address new ways to help younger people in managing their anxiety. We also hope this study will help us understand future social behaviors, from both a psychological and sociological perspective.

It is likely that the consequences of the pandemic will be pervasive and prolonged, and we still do not know what is going to happen in the near future, so it would be interesting to monitor attitudes toward touch in the coming months and years, reporting on their evolution. It would also be interesting to further study the attitudes of older people, trying to understand why they feel less anxious even when they are the most exposed to mortality. Understanding their attitudes more deeply could help keep them safer, motivating them to conduct themselves conscientiously.

## Data Availability Statement

The datasets presented in this study can be found in online repositories. The names of the repository/repositories and accession number(s) can be found below: https://github.com/M-Pass/TouchAvoidance_Covid/.

## Ethics Statement

The studies involving human participants were reviewed and approved by the Ethics Committee of Policlinico “Paolo Giaccone,” in Palermo, Italy. The patients/participants provided their written informed consent to participate in this study.

## Author Contributions

LC and LR collected the data. MP and LC organized the database. MP performed the statistical analysis. All authors contributed to conception and design of the study, wrote the first draft of the manuscript, and contributed to manuscript revision, read, and approved the submitted version.

## Conflict of Interest

The authors declare that the research was conducted in the absence of any commercial or financial relationships that could be construed as a potential conflict of interest.

## Publisher’s Note

All claims expressed in this article are solely those of the authors and do not necessarily represent those of their affiliated organizations, or those of the publisher, the editors and the reviewers. Any product that may be evaluated in this article, or claim that may be made by its manufacturer, is not guaranteed or endorsed by the publisher.

## Footnotes

1 In the pre-pandemic sample, geographical region was associated with touch avoidance toward family members [lower in the South than in the North; *t*(311) = 4.05, *p* < 0.001]. In the post-pandemic sample, geographical region was associated with touch avoidance toward family members [lower in the South than in the North; *t*(671) = −4.52, *p* < 0.001], touch avoidance toward strangers [higher in the South than in the North; *t*(671) = 4.42, *p* < 0.001], stress [higher in the South than in the North; *t*(671) = 4.05, *p* < 0.001], and anxiety [higher in the South than in the North; *t*(671) = 5.20, *p* < 0.001].
