# Supplementary material for: Changes in Touch Avoidance, Stress, and Anxiety During the COVID-19 Pandemic in Italy
Source: Front Psychol. 2022 Jul 22;13:854110. doi: 10.3389/fpsyg.2022.854110 (PMC9354952; doi:10.3389/fpsyg.2022.854110)
Supplement: Supplementary file 1 [file Data_Sheet_1.docx]

Table S1.

Study 1 descriptive statistics.

|  | Prior to the pandemic | | | | During the pandemic | | | |
| --- | --- | --- | --- | --- | --- | --- | --- | --- |
| (Sub)scale | Mean | SD | Skewness | Kurtosis | Mean | SD | Skewness | Kurtosis |
| TAQ Partner | 1.86 | .44 | .41 | -.83 | 1.85 | .68 | 1.88 | 4.16 |
| TAQ Family | 2.55 | 1.13 | .34 | -1.04 | 2.63 | 1.14 | .28 | -.99 |
| TAQ Same Sex | 1.94 | .74 | .98 | .41 | 1.71 | .62 | .36 | -1.12 |
| TAQ Opposite Sex | 2.01 | .67 | .87 | .65 | 1.96 | .81 | .49 | -.69 |
| TAQ Stranger | 3.28 | .64 | .14 | -.98 | 2.49 | .85 | .18 | -.84 |
| MSP | 1.71 | .35 | .99 | 1.59 | 1.83 | .48 | .58 | -1.00 |
| STAI | 2.42 | .43 | .50 | .08 | 2.45 | .65 | .66 | -.68 |

Table S2.

Study 2 descriptive statistics.

|  | Prior to the pandemic | | | | During the pandemic | | | |
| --- | --- | --- | --- | --- | --- | --- | --- | --- |
| (Sub)scale | Mean | SD | Skewness | Kurtosis | Mean | SD | Skewness | Kurtosis |
| TAQ Partner | 1.94 | .60 | .94 | .45 | 1.94 | .73 | .83 | 1.01 |
| TAQ Family | 2.65 | .96 | .23 | -.57 | 2.86 | 1.02 | .09 | -.81 |
| TAQ Same Sex | 2.07 | .74 | .89 | .92 | 1.92 | .84 | .85 | .26 |
| TAQ Opposite Sex | 2.16 | .74 | .82 | .71 | 2.02 | .87 | .65 | -.23 |
| TAQ Stranger | 2.62 | .80 | .25 | -.18 | 2.83 | .90 | .16 | -.50 |
| MSP | 1.76 | .40 | .20 | -.55 | 2.13 | .58 | .52 | -.43 |
| STAI | 2.60 | .53 | .90 | .50 | 2.77 | .64 | .16 | -.79 |
| MAC-RF1 | - | - | - | - | 1.50 | 1.12 | .35 | -.87 |
| MAC-RF2 | - | - | - | - | 3.19 | 1.13 | -.17 | -.89 |
| MAC-RF3 | - | - | - | - | 2.10 | .91 | .64 | -.06 |
| MAC-RF4 | - | - | - | - | 2.31 | 1.11 | .50 | -.66 |


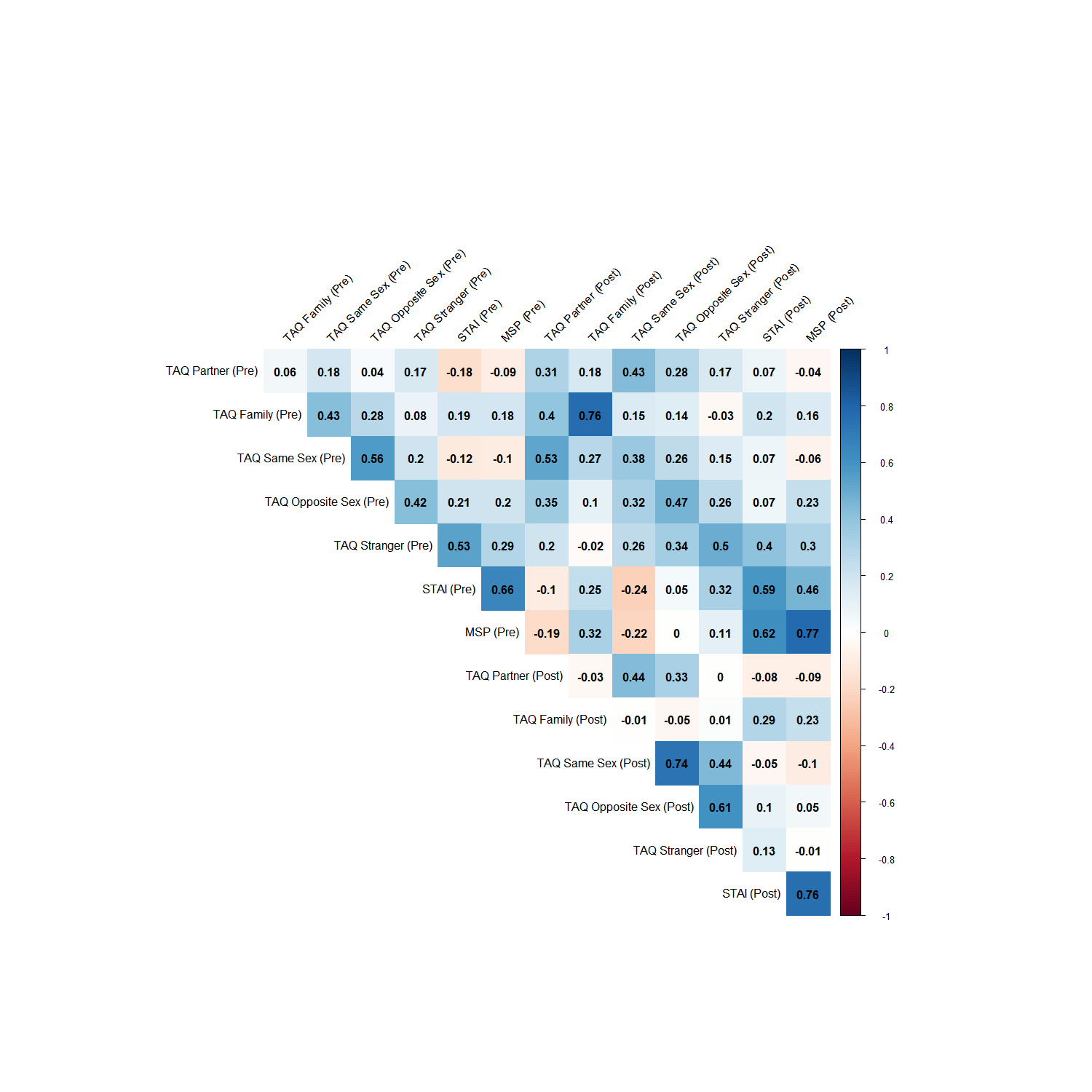


Figure S1.

Correlogram for Study 1.


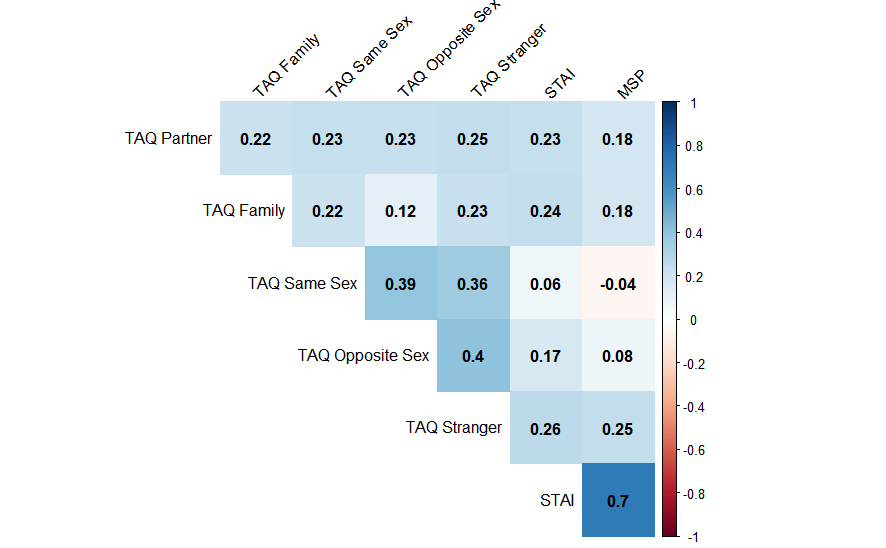


Figure S2.

Correlogram for Study 2, pre-pandemic measures.


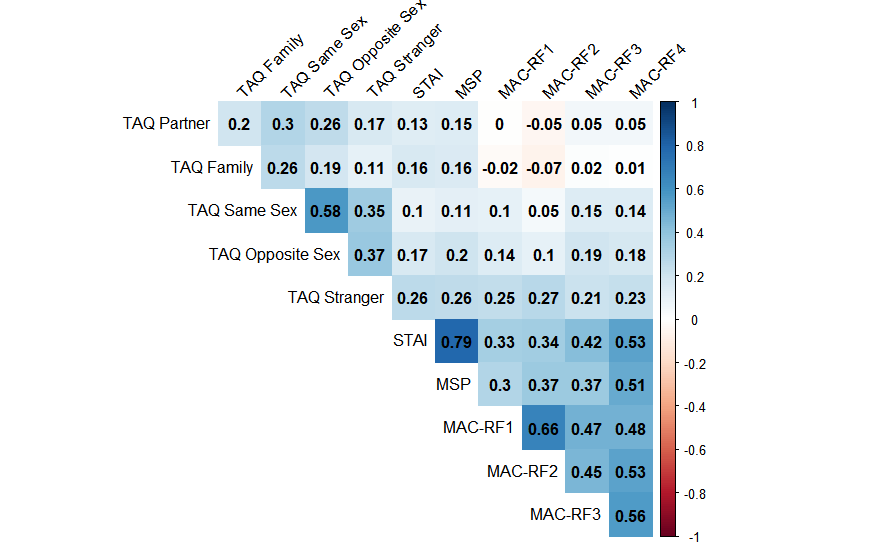
tr

Figure S3.

Correlogram for Study 2, measures taken during the pandemic.
